# Efficacy and safety of ramucirumab treatment in patients with advanced colorectal cancer: A protocol for systematic review and meta analysis

**DOI:** 10.1097/MD.0000000000020618

**Published:** 2020-06-12

**Authors:** Man Ju, Honggang Cheng, Kai Qu, Xiangqian Lu

**Affiliations:** a Department of Anus & Intestine Surgery; b Department of Gastrointestinal Surgery, Liaocheng People's Hospital, Liaocheng, Shandong Province; c Department of Hepatobiliary Surgery, The First Affiliated Hospital of Xi’an Jiaotong University, Xi’an, Shaanxi Province; d Department of Radiotherapy, Liaocheng People's Hospital, Liaocheng, Shandong Province, China.

**Keywords:** colorectal cancer, efficacy, meta-analysis, ramucirumab, safety

## Abstract

**Background:**

vascular endothelial growth factor receptor 2 (VEGFR-2) has an important role in colorectal cancer pathogenesis and progression. The aim of our study is to provide a protocol for assessing the efficacy and safety of ramucirumab (a monoclonal antibody VEGFR-2 antagonist) for the treatment of advanced colorectal cancer.

**Methods:**

The systematic review will be reported according to the preferred reporting items for systematic reviews and meta-analyses protocols. Relevant randomized controlled trials were searched from PubMed, Cochrane Library, Web of Science, Excerpt Medica Database, China National Knowledge Infrastructure, and Wanfang Database. Papers in English or Chinese published from their inception to February 2020 will be included without any restrictions.

Study selection and data extraction will be performed independently by 2 investigators. The clinical outcomes including overall response rate, complete response rate (disease control rate), overall survival, progression-free survival, quality of life, immune function, and adverse events, were systematically evaluated. Review Manager 5.3 and Stata 14.0 were used for data analysis, and a fixed or random-effect model of meta-analysis will be used depending upon the heterogeneity observed between studies. Subgroup analysis will be carried out depending on the availability of sufficient clinical data.

**Results and Conclusion:**

The findings of this systematic review and meta-analysis will be published in a peer-reviewed journal, and provide more evidence-based guidance in clinical practice.

**PROSPERO registration number:**

CRD42020165683.

## 1 Introduction

Colorectal cancer (CRC) is the second most frequent cause of cancer-related death and caused 861,700 deaths worldwide in 2018.^[1,2]^ In recent years, the incidence of CRC has significantly raised with about 1.8 million new cases every year.^[1,2]^ CRC is often diagnosed in an advanced stage due to hiding of clinical symptom.^[3]^ It is demonstrated that approximately 25% of CRC patients with metastases are diagnosed initially and nearly 50% of them will develop metastases afterwards.^[3,4]^ Patients with metastatic disease have a poor prognosis with a 5-year survival rate of only 13.1%.^[4]^ Surgery, radiotherapy and chemotherapy are the most widely used therapeutic methods for CRC.^[5]^ However, many researchers reported that these conventional treatments was not able to completely eradicate small lesions and metastatic cells, which may raise the probability of cancer recurrence.^[5]^ Thus, more effective and safer treatments were urgently required.^[6,7]^

Angiogenesis plays a central role in tumor growth and metastasis.^[4,8]^ Tumors require a vascular supply to grow that is achieved via the expression of pro-angiogenic growth factors, including members of the vascular endothelial growth factor (VEGF) family of ligands.^[4,9]^ Tumor progression and poor prognosis in numerous tumor types, including CRC, has been associated with the overexpression of VEGF. VEGF ligands mediate their angiogenic effects via several different receptors.^[4,10]^ VEGFR2, expressing in vessel endothelial cells, is the main receptor for the angiogenesis and responsible for proliferation, migration of endothelial cells.^[4]^ Preclinical studies have demonstrated that blockade of the VEGF-A/vascular endothelial growth factor receptor 2 (VEGFR-2) interaction inhibits tumor angiogenesis and growth, rendering it a promising approach in anticancer treatments.^[11]^ Ramucirumab (Cyramza; IMC-1121B; LY3009806; Lilly Oncology) is a fully humanized immunoglobulin G1 monoclonal antibody that binds with high affinity to the VEGFR-2 extracellular domain, blocking all VEGF ligands from binding to this therapeutically validated target.^[12–14]^ As such, ramucirumab has the potential capacity to inhibit multiple activities initiated by VEGF activation of VEGFR-2.^[12–16]^

Due to the improvement of overall survival (OS) and progression free survival (PFS) reported by the phase II/III clinical trial, ramucirumab treatment has been widely explored in the treatment of solid tumors,^[12,15,17–19]^ and approved by the US Food and Drug Administration for the treatment metastatic CRC in 2015.^[12]^ Many studies showed an obvious advantage for ramucirumab combined with conventional medicines in both OS and PFS of metastatic CRC patients.^[4,12]^ Despite the intensive clinical studies, its clinical efficacy was still not systematically evaluated. We are prepared to summarize the efficacy and adverse events of ramucirumab treatment of CRC at advanced stages through the meta-analysis, in order to provide scientific reference for the design of future clinical trials.

## 2 Study aim

The aim of this meta-analysis was to systematically evaluate the efficacy and safety of ramucirumab mediated therapy for the treatment of advanced CRC.

## 3 Methods

The protocol of our meta-analysis will be reported according to preferred reporting items for systematic review and meta-analysis protocols guidelines.^[20]^ Our protocol has been registered on the International Prospective Register of Systematic Review. The registration number was CRD42020165683 (Available from: https://www.crd.york.ac.uk/prospero/display_record.php?ID=CRD42020165683). This meta-analysis is a secondary research which based on some previously published data. Therefore, the ethical approval or informed consent was not required in this study.

### 3.1 Data sources

Six electronic databases including Cochrane Library, Web of Science, PubMed, Excerpt Medica Database, China National Knowledge Infrastructure, and Wanfang Database will be systematically searched for eligible studies from their inception to February 2020. Language is limited with English and Chinese.

### 3.2 Search strategy

To perform a comprehensive and focused search, experienced systematic review researchers will be invited to develop a search strategy. The plan searched terms are as follows: “colorectal cancer” or “colorectal neoplasm” or “colorectal carcinoma” or “colorectal tumor” or “colon cancer” or “colon neoplasm” or “colon carcinoma” or “colon tumor” or “rectal cancer” or “rectal neoplasm” or “rectal carcinoma” or “rectal tumor” or “CRC” or “CC” or “RC” and “ramucirumab” or “IMC1121B” or “LY3009806” or “Cyramza” et al. An example of search strategy for PubMed database shown in Table 1 will be modified and used for the other databases.

**Table 1:**
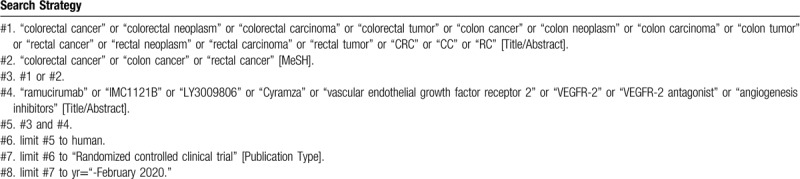
Searching strategy in PubMed.

### 3.3 Eligibility criteria

#### 3.3.1 Types of studies

All available randomized controlled trials (RCTs) that investigated the efficacy and safety of ramucirumab treatment in patients diagnosed with advanced CRC will be included in this systematic review. Articles without sufficient available data, noncomparative studies, non-RCTs, literature reviews, meta-analysis, meeting abstracts, and case reports will be excluded.

#### 3.3.2 Participants

Patients must be cytologically or pathologically confirmed as having CRC at a clinically advanced stage. There will be no limitations on age, gender, racial and region. Patients with other malignancies or nonprimary CRC are not included.

#### 3.3.3 Interventions

CRC patients treated with conventional medicines and ramucirumab targeted therapy will be included. The dose and administered frequency of ramucirumab are not restricted.

#### 3.3.4 Comparisons

CRC patient treated with the same conventional medicine as intervention group in the same original study.

#### 3.3.5 Language

Studies published in English and Chinese will be included.

### 3.4 Outcomes

#### 3.4.1 Primary outcomes

The primary outcomes will be the therapeutic effects of treatment according to Response Evaluation Criteria in Solid Tumors 1.1.^[21]^

(1) Overall response rate and disease control rate;
(2) OS (which is defined as the time from the date of randomization to death from any cause);
(3) PFS (which is defined as the time from the date of randomization until disease progression or death).

#### 3.4.2 Secondary outcomes

Secondary outcomes will include:

(1) immune function evaluation;
(2) quality of life as evaluated by Karnofsky score, and
(3) treatment-related adverse effects assessment.

#### 3.4.3 Outcome follow-up periods

Early and durable response will be recorded among included studies. All time points will be considered due to the anticipated variability in follow-up. The details of the follow-up period will also be recorded for all studies.

### 3.5 Study selection and management

**Figure 1:**
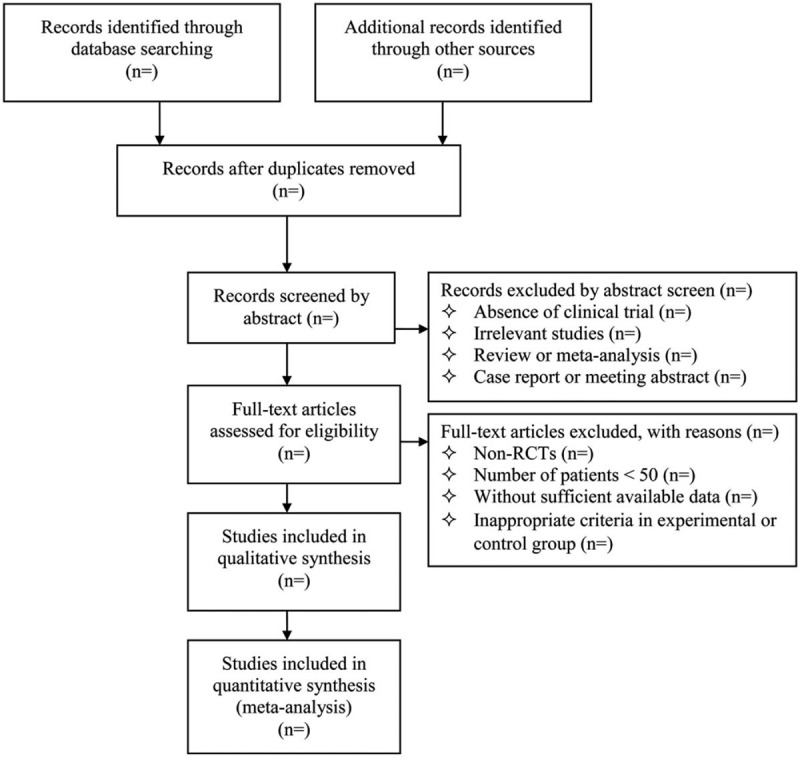
Study selection process for the meta-analysis. RCTs = randomized controlled trials.

Endnote X7 software will be used for literature managing and records searching. Two authors (Ju M and Cheng HG) will be reviewed independently to identify potential trials by assessing the titles and abstracts. The full text of all relevant trials will be further evaluated to make sure eligible trials. Any conflict will be resolved through discussion with a third reviewer (Qu K). A preferred reporting items for systematic review and meta-analysis-compliant flow diagram (Fig. 1) will be used to describe the selection process of eligible literatures.

### 3.6 Data extraction and management

Two reviewers (Ju M and Cheng HG) will be responsible for the data extraction independently according to the Cochrane Handbook for Systematic Reviews of Intervention. The following data will be extracted from eligible literatures: the first author, year of publication, country of study, participants (sample size, tumor-node-metastasis stage, age, gender, inclusion and exclusion criteria, etc), details of all experimental and control interventions regimen (dosage of ramucirumab, administration route, duration of treatment, follow-up time, etc), outcomes (overall response rate, disease control rate, OS, PFS, quality of life, immune function and adverse effects). For survival outcomes, Hazard ratios with corresponding 95% confidence intervals will be extracted from trials or be estimated from Kaplan–Meier survival curves by established methods.^[22]^ Any disagreements will be resolved by discussion, and a third reviewer (Qu K) will make the final decision. Excluded studies and the reasons for exclusion will be listed in a table.

### 3.7 Dealing with missing data

We will attempt to contact the authors to request the missing or incomplete data. If those relevant data are not acquired, they will be excluded from the analysis.

### 3.8 Risk of bias of individual study

The quality of the included RCTs will be assessed independently by 2 investigators (Ju M and Cheng HG) in terms of sequence generation, allocation concealment, blinding, incomplete outcome data, selective reporting, and other bias, according to the guidance of the Cochrane Handbook for Systematic Review of Interventions.^[23,24]^ Evidence quality will be classified as low risk, high risk, or unclear risk of bias in accordance with the criteria of the risk of bias judgment. Any disagreements will be resolved via discussion with a third researcher (Qu K).

### 3.9 Data synthesis

Statistical analyses will be performed using Review Manager 5.3 (Nordic Cochran Centre, Copenhagen, Denmark) and Stata 14.0 (Stata Corp., College Station, TX) statistical software. The outcomes were mainly represented by risk ratio with its 95% confidence intervals. A 2-tailed *P*-value < .05 was considered statistically significant. Cochrane *Q*-test and *I*^2^ statistics were used to assess heterogeneity between studies; *P* < .1 or *I*^2^ > 50% indicates statistical heterogeneity.^[25]^ A fixed effect model will be used to calculate the outcomes when statistical heterogeneity is absent; otherwise, the random effects model was considered according to the DerSimonian and Laird method.^[26]^

### 3.10 Publication bias analyses

We will detect publication biases and poor methodological quality of studies using funnel plots if 10 or more studies are included in the meta-analysis. Begg and Egger regression test will be utilized to detect the funnel plot asymmetry.^[27–29]^ If publication bias existed, a trim-and-fill method should be applied to coordinate the estimates from unpublished studies, and the adjusted results were compared with the original pooled RR.^[30,31]^

### 3.11 Assessment of heterogeneity

#### 3.11.1 Sensitivity analysis

Sensitivity analysis was conducted to explore an individual study's influence on the pooled results by deleting 1 single study each time from pooled analysis. A summary table will report the results of the sensitivity analyses.

#### 3.11.2 Subgroup and meta-regression analysis

If the data are available and sufficient, subgroup and meta-regression analysis will be conducted to explore the source of heterogeneity with respect to age, gender, region, tumor stage, sample sizes, follow-up period, chemotherapy regimens, and types of involved studies.

### 3.12 Dissemination plans

We will disseminate the results of this systematic review by publishing the manuscript in a peer-reviewed journal or presenting the findings at a relevant conference.

## 4 Discussion

Current treatment methods for CRC only have a modest survival benefit. With the development and clinical application of molecule-targeted drugs, the molecule-targeted treatment of tumors has been widely accepted.^[32–35]^ The agents used in targeted therapy can precisely identify and attack certain type of cancer cells based on mutations of genes and proteins, with little damage for normal cells.^[32–35]^ As an effective molecule-targeted agent, ramucirumab has been widely used for the treatment of diverse malignant tumors worldwide.^[12,15,17–19]^

### 4.1 Strengths and limitations of this study

Even though there was statistical analysis of published clinical trials, the exact therapeutic effects of ramucirumab treatment were still not systematically investigated. This systematic review may provide helpful evidence for clinicians, and patients who use ramucirumab for the treatment of advanced CRC.

The systematic review will also have some limitations. There may be a language bias with the limitation of English and Chinese studies. Clinical heterogeneity may exist for different tumor stage and ages of CRC patients, dosage of ramucirumab, and duration of treatment.

## Author contributions

**Conceptualization:** Xiangqian Lu and Kai Qu.

**Investigation:** Xiangqian Lu, Man Ju, and Honggang Cheng.

**Methodology:** Xiangqian Lu, Man Ju, and Honggang Cheng.

**Project administration:** Xiangqian Lu.

**Supervision:** Xiangqian Lu and Kai Qu.

**Funding acquisition:** Kai Qu.

**Writing – original draft:** Man Ju and Honggang Cheng.

**Writing – review and editing:** Xiangqian Lu and Man Ju.
